# Implementing COVID-19 Simulation Training for Anesthesiology Residents

**DOI:** 10.15766/mep_2374-8265.11215

**Published:** 2022-01-31

**Authors:** Bryant E. Hong, Christine C. Myo Bui, Yue Ming Huang, Tristan Grogan, Victor F. Duval, Maxime Cannesson

**Affiliations:** 1 Resident, Department of Anesthesiology and Perioperative Medicine, University of California, Los Angeles, David Geffen School of Medicine; 2 Associate Clinical Professor and Associate Residency Program Director, Department of Anesthesiology and Perioperative Medicine, University of California, Los Angeles, David Geffen School of Medicine; 3 Interim Executive Director, UCLA Simulation Center; Associate Adjunct Professor, Department of Anesthesiology and Perioperative Medicine, University of California, Los Angeles, David Geffen School of Medicine; 4 Principal Statistician, Department of Medicine Statistics Core, University of California, Los Angeles, David Geffen School of Medicine; 5 Anesthesiology Liaison and Lead Instructor, UCLA Simulation Center; Associate Clinical Professor, Department of Anesthesiology and Perioperative Medicine, University of California, Los Angeles, David Geffen School of Medicine; 6 Professor and Chair, Department of Anesthesiology and Perioperative Medicine, University of California, Los Angeles, David Geffen School of Medicine

**Keywords:** High-Fidelity Simulation Training, COVID-19, Personal Protective Equipment, Airway Management, Anesthesiology, Simulation, Editor's Choice

## Abstract

**Introduction:**

During the COVID-19 pandemic, anesthesiology residents faced increased risk of exposure to SARS-CoV-2 while performing aerosolizing procedures. We developed an airway simulation on the out-of-operating-room management of COVID-19 patients.

**Methods:**

A 90-minute simulation focused on caring for a 45-year-old COVID-19 patient provided training in donning and doffing personal protective equipment, intubation, management of postinduction hypotension, management of ICU ventilators, treatment strategies for acute respiratory distress syndrome (ARDS), interpersonal communication, and resource management. Presimulation, postsimulation, and 3-months postsimulation questionnaires measured changes in confidence, knowledge, and clinical practice. Statistical analysis was completed using related-samples Wilcoxon signed rank tests.

**Results:**

Twenty-four residents participated in the simulation. Questionnaire response rates were 100% presimulation and postsimulation and 88% 3-months postsimulation. Confidence scores (1 = *not at all,* 5 = *extremely*) improved with donning and doffing personal protective equipment (from 3.0 to 4.1, *p* < .001), ARDS management (from 3.1 to 4.0, *p* < .001), and COVID-19 airway management (from 2.8 to 4.0, *p* < .001). Correct answers on 10 knowledge questions increased significantly between presimulation and postsimulation (from 5.1 to 9.0, *p* < .001) but not between presimulation and 3-months postsimulation (from 5.1 to 5.8, *p* = .27). All participants who cared for COVID-19 patients at 3 months agreed or strongly agreed that their current management of COVID-19 patients was directly influenced by the simulation session (*M* = 4.4).

**Discussion:**

This simulation is a safe, effective method of providing the experiential training necessary to care for actual COVID-19 patients during an active pandemic.

## Educational Objectives

By the end of this activity, learners will be able to:

1. Formulate and implement a plan for treating acute respiratory distress in a COVID-19 patient within a nonoperative setting.
2. Manage a COVID-19 patient with acute respiratory distress syndrome.
3. Demonstrate teamwork and interpersonal communication skills with team members, the patient, and the patient's family member.

## Introduction

Severe acute respiratory syndrome coronavirus 2 (SARS-CoV-2), the virus that leads to coronavirus disease 2019 (COVID-19), overwhelmed hospital systems throughout 2020. Health care providers (HCPs) were on the front lines of patient contact, and concerns developed over insufficient patient testing, nosocomial infection, and limitations in the availability of and training with personal protective equipment (PPE).^1,2^ One early single-site prospective cohort study showed that HCPs had a higher prevalence of SARS-CoV-2 infection (7.3%) compared to non-HCPs (0.4%).^3^ Anesthesia providers specifically faced further risk from work-related exposure to airborne and droplet pathogens while performing aerosolizing procedures.^4,5^ Later studies revealed a lower prevalence of SARS-CoV-2 antibodies among HCPs who wore face masks, with the lowest seropositivity rates in those who wore N95 respirators or powered air-purifying respirators (PAPRs), reinforcing the success of universal PPE precautions.^6–8^

As part of an institution-wide response to the COVID-19 pandemic, the University of California, Los Angeles (UCLA), Department of Anesthesiology and Perioperative Medicine (DAPM) formed a task force in March 2020 to develop guidelines related to COVID-19 surge planning, perioperative patient testing, and HCP protection. UCLA postgraduate year four (PGY 4) anesthesiology residents reported limited exposure to PPE and COVID-19 patients, yet these were also the same providers most likely to be called upon during a surge. Whereas many simulation centers closed during the pandemic, our simulation center remained open with COVID-19 safety precautions in place, allowing us to continue with PPE testing and training.

Between May and June 2020, the UCLA DAPM developed and implemented a high-fidelity COVID-19 airway simulation for residents on the out-of-operating-room management of COVID-19 patients in respiratory distress. The goals of our educational project were to prepare PGY 4 anesthesiology residents for COVID-19 patient exposure during an active pandemic, illustrate the importance of simulation training during an active pandemic, and measure the impact of simulation training on PGY 4 anesthesiology residents’ confidence, knowledge, and clinical management of actual COVID-19 patients.

## Methods

### Development

We created a simulation scenario for the out-of-operating-room airway management of a COVID-19 patient (Appendix A). This scenario was developed by a team of UCLA anesthesiologists with roles as both clinical faculty and simulation instructors and the UCLA simulation education team. We identified learning objectives based on skills likely to be utilized by anesthesiologists during the COVID-19 pandemic. Skills included donning and doffing of PPE, intubation of COVID-19 patients, management of postinduction hypotension, management of ICU ventilators, treatment strategies for acute respiratory distress syndrome (ARDS), teamwork and interpersonal communication, and resource management. Learning objectives were integrated into either the simulation or the debrief session.

The simulation session was 90 minutes: 20 minutes of orientation, 30 minutes of simulation, and 40 minutes of debriefing. The simulation sessions took place from May to June 2020. Twenty-four PGY 4 anesthesiology residents were chosen for training because of their likelihood of intubating COVID-19 patients on call teams or in practice after graduation. Prior to the simulation, all residents participated in an N95 respirator fitting, a PAPR demonstration (observation only), and a perioperative donning and doffing training session with N95 respirators, eye protection, head coverings, gowns, and gloves.

### Consent

Informed written consent to record and collect data for research was obtained from each participant prior to starting the simulation (UCLA IRB#11-001330).

### Equipment/Environment

This live training took place at the UCLA Simulation Center. The ICU simulation room included a full-body manikin (Laerdal SimMan 3G), a Servo-i ventilator, and an ASL 5000 breathing simulator (IngMar Medical) that was preconfigured to simulate ARDS. The anteroom included PPE, airway equipment, anesthetic medication, mirrors, and cognitive aids for donning and doffing developed by the UCLA COVID-19 Task Force (Appendix B). From a control room, instructors observed the participants, controlled the manikin, and manipulated the breathing simulator.

### Personnel

We designed our simulation to accommodate 24 PGY 4 residents working in two-person teams. One simulation specialist acted as the embedded ICU nurse, and another played the voice of the patient and the patient's family member. An anesthesiologist instructor acted as the ICU attending and safety monitor. An anesthesiology resident observed and collected data with mentorship from an education specialist. Four instructors in total facilitated the debriefing. All instructors had previously completed formal simulation education training, which focused on debriefing techniques and scenario development. All personnel wore appropriate PPE and followed COVID-19 safety protocols. Simulation center staff were trained on PAPR by the Director of Emerging Infectious Disease Preparedness.

### Implementation

Anesthesiology residents were relieved from scheduled responsibilities for the simulation. Upon arrival at the simulation center with face masks, they had their temperature checked, performed hand hygiene, and sat socially distanced in a debriefing room. A simulation specialist provided them with the case stem and patient history of present illness. The participants then completed a presimulation questionnaire (Appendix C).

At the start of the simulation, the participants were called to evaluate a 45-year-old COVID-19 patient in respiratory distress. Participants donned their choice of either an N95 respirator or a PAPR in the anteroom. Cognitive aids for donning and doffing were posted as references. The ICU attending served as a safety monitor and offered real-time instruction during the donning and doffing process. In the ICU room, participants encountered an anxious COVID-19 patient presenting with respiratory failure. An embedded ICU nurse assisted the participants in the room. The sequence of events required participants to communicate with the ICU nurse and the patient, induce and intubate the patient, manage postinduction hypotension, and manage the ICU ventilator in a patient with ARDS. Once the patient was stabilized, the participants doffed their PPE in two stages—first in the patient's room, then in the anteroom. The scenario ended after participants provided a verbal sign-out to the ICU attending and updated the patient's family member over the phone.

### Assessment

Simulation instructors used a 20-item critical action checklist to evaluate resident performance during the simulation (Appendix A). Simulations were video recorded and subsequently reviewed by a single investigator (Bryant E. Hong). Point values were rewarded for performing actions correctly (1 point), in order (1 point), and without prompting (1 point), for 3 total points. UCLA COVID-19 donning and doffing guidelines were used as a reference for donning and doffing (Appendix B). National Heart, Lung, and Blood Institute (NHLBI) ARDSnet protocol was used as a reference for lung protective ventilation.^9^

Three questionnaires developed for presimulation, postsimulation, and 3-months postsimulation assessed effectiveness of training by measuring for changes in confidence, knowledge, and clinical practice. These metrics were based on Kirkpatrick's four levels of evaluation.^10^ Improvement in confidence (level one) was measured presimulation and postsimulation using questions based on a 5-point Likert scale (1 = *not at all,* 5 = *extremely*). Improvement in knowledge (level two) was measured using 10 multiple-choice questions given presimulation, postsimulation, and 3-months postsimulation. Authors with clinical and educational expertise developed the questions through an iterative consensus process and covered potential knowledge gaps residents might encounter while intubating COVID-19 patients. The content of the questions included steps for PPE donning and doffing, minimum procedure-specific requirements for PPE, filtration efficiency of N95 respirators versus PAPRs, NHLBI ARDSnet protocol, and prone positioning (Appendix C). Changes in clinical practice (level three) were measured using self-reported questions in the 3-months postsimulation questionnaire. Patient outcomes (level four) were not evaluated.

We distributed questionnaires through Qualtrics. Responses were deidentified prior to analysis. Confidence-based questions were statistically compared presimulation versus postsimulation, whereas knowledge-based questions were compared presimulation versus postsimulation and presimulation versus 3-months postsimulation. Due to the small sample size, we were not sure that the normality assumption of the paired-sample *t* test was satisfied. Therefore, we opted for the nonparametric related-samples Wilcoxon signed rank test, which did not require that assumption. We also presented the difference between time periods with 95% confidence intervals. Statistical analyses were conducted using IBM SPSS V26, and *p* values less than .05 were considered statistically significant.

### Debriefing

Debrief sessions were led by anesthesiology instructors, who used established techniques to guide reflective learning and discussion.^11,12^ Debriefing topics included UCLA recommendations for airway management of COVID-19 patients, donning and doffing of PPE, ARDSnet protocol, prone positioning, a hands-on component with PAPRs, teamwork, and interpersonal communication. The debriefing ended with an open discussion and time for feedback and questions. Participants were then asked to complete the postsimulation questionnaire (Appendix C).

## Results

Twenty-four PGY 4 anesthesiology residents participated in one of 12 total simulation sessions. Response rates were 100% for the presimulation and postsimulation questionnaires and 88% (21 of 24) for the 3-months postsimulation questionnaire. Three participants who were lost to follow-up at 3 months were removed from knowledge assessment analysis. Prior to the simulation, 42% (10 of 24) of the residents did not have any exposure to COVID-19 patients. Of the residents who had exposure to COVID-19 patients, 79% (11 of 14) had taken care of three or fewer COVID-19 patients. PPE utilization amongst the residents was also limited. Only two participants reported experience with donning and doffing a PAPR prior to the simulation.

The 20-item critical action checklist used to evaluate resident performance during the simulation guided the debriefing discussions. Twenty videos were analyzed, with four videos missing due to technical difficulties. Overall, the participants performed well, receiving full points or near-full points for most actions. Critical steps that were occasionally missed included inadequate preoxygenation, failure to prepare for a possible difficult airway, and failure to verify endotracheal tube positioning after intubation (Table 1). These critical steps amongst others are addressed under Anticipated Management Mistakes in Appendix A.

**Table 1.**
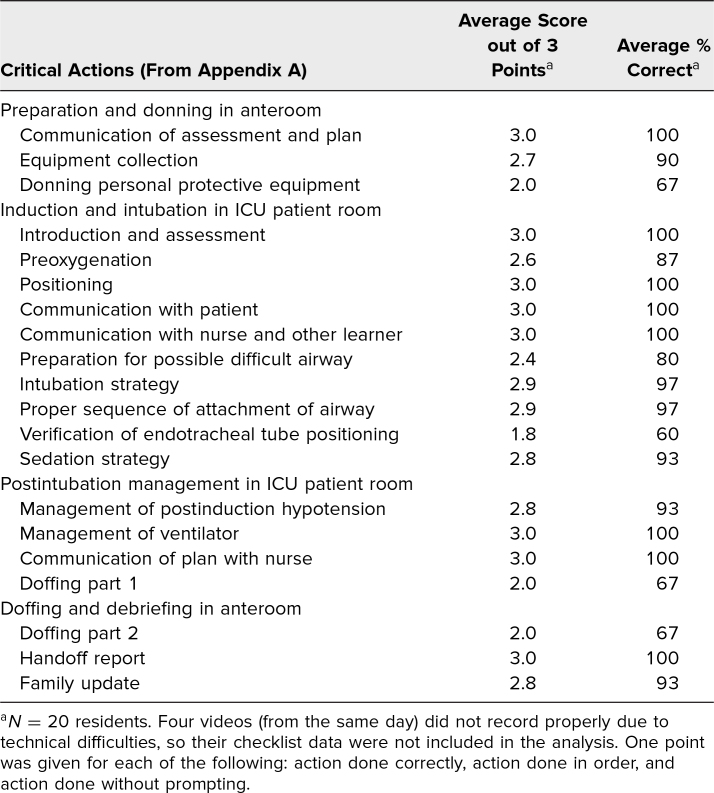
Critical Actions Checklist Results

All participants required assistance with donning and doffing even with the presence of cognitive aids. Twenty participants donned and doffed with PAPRs, while four donned and doffed with N95 respirators. Most were unfamiliar with PAPRs, sometimes leading to inappropriate handling of PAPRs, inadequate equipment checks, and time-consuming periods of donning and doffing.

At 3-months postsimulation, 10 of 21 participants reported that they had cared for a COVID-19 patient. All 10 participants either agreed or strongly agreed that their current management of COVID-19 patients was directly influenced by the simulation session (*M* = 4.4 of 5), and 90% of participants either agreed or strongly agreed that principles of PPE taught during the simulation session were similar to their institution's current practices (*M* = 4.1 of 5). Participants who wore a PAPR in the simulation reported that the simulation was more helpful than the PAPR demonstration provided by the UCLA anesthesiology department (*M* = 5.0 of 5; Table 2).

**Table 2.**
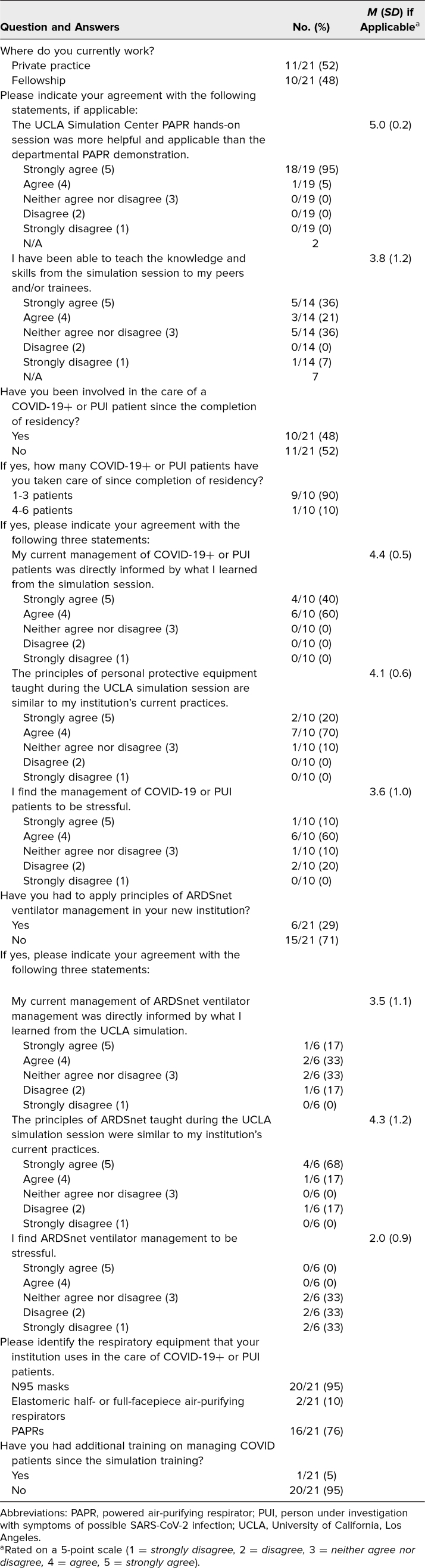
Three-Month Follow-up Questionnaire Results

Confidence improved immediately after simulation training. Residents reported higher confidence with donning and doffing of PPE (from 3.0 to 4.1 of 5, *p* < .001), ARDS management (from 3.1 to 4.0 of 5, *p* < .001), and COVID-19 airway management (from 2.8 to 4.0 of 5, *p* < .001; Table 3).

**Table 3.**
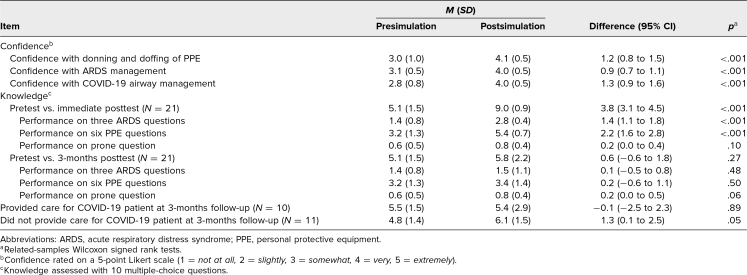
Questionnaire Results

Knowledge assessment scores showed statistical improvement postsimulation (from 5.1 to 9.0 of 10, *p* < .001, *N* = 21) but not at 3-months postsimulation (from 5.1 to 5.8 of 10, *p* = .27, *N* = 21; Table 3; Figure). Stratifying the data by exposure to COVID-19 patients in clinical practice at 3 months showed a statistically significant improvement in those who did not provide care for COVID-19 patients (from 4.8 to 6.1 of 10, *p* = .05, *N* = 11) but not in those who provided care for COVID-19 patients (from 5.5 to 5.4 of 10, *p* = .89, *N* = 10). Stratifying the data based on question type did not result in any statistically significant results (Table 3).

**Figure.**
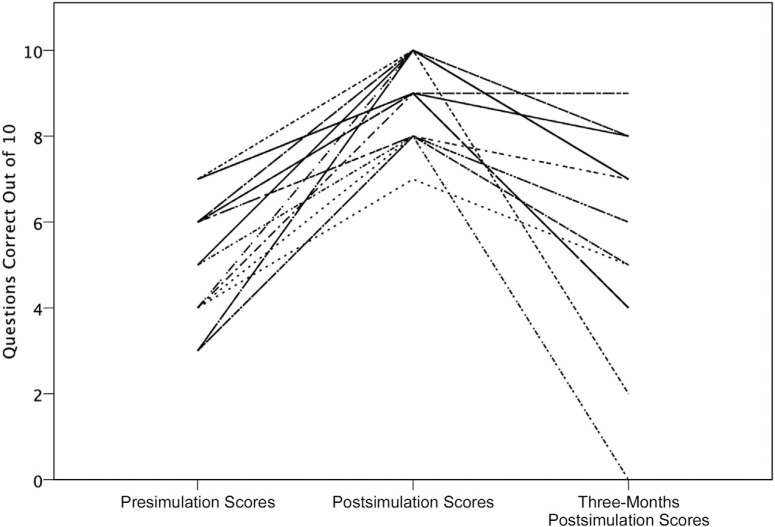
Knowledge assessment scores of 21 participants presimulation versus postsimulation versus 3-months postsimulation. Each line is a separate participant and may represent two or more participants who had identical scores. Mean assessment scores of participants were 5.1 out of 10 (*SD* = −1.5) presimulation versus 9.0 out of 10 (*SD* = −0.9) postsimulation versus 5.8 out of 10 (*SD* = −2.2) 3-months postsimulation.

Only six participants reported using ARDSnet principles in clinical practice. There was significant variability when they were asked if their current ARDS ventilator management was influenced by the simulation session (*M* = 3.5 of 5, *SD* = 1.1) and whether ARDSnet principles taught during the UCLA simulation session were similar to their institution's current practices (*M* = 4.3 of 5, *SD* = 1.2; Table 2).

We also asked about stress on the postsimulation and 3-months postsimulation questionnaires. Although most participants did not find the simulation stressful (*M* = 1.6 of 5), many participants who had exposure to COVID-19 patients after the simulation reported higher stress with the care of COVID-19 patients in clinical practice (*M* = 3.6 of 5). Most did not find ARDSnet ventilator management to be stressful (*M* = 2.0 of 5; Table 2).

## Discussion

Simulation was instrumental in training providers during the 2003 severe acute respiratory syndrome outbreak,^13,14^ the 2012 Middle East respiratory syndrome outbreak,^15^ and the 2014 Ebola outbreak in West Africa.^16–18^ Earlier studies prior to and during the COVID-19 pandemic similarly utilized simulation to teach donning and doffing of PPE,^19,20^ to prepare for airway management,^21^ and to improve institutional COVID-19 protocols.^22^

The use of high-fidelity simulation to train anesthesiology residents during the COVID-19 pandemic provided critical hands-on experience with PAPRs. Prior to the simulation, all anesthesiology residents attended a departmental PAPR demonstration as passive learners. The simulation was the first time that 18 residents had practiced PAPR donning and doffing with live coaching from an experienced observer. Most residents reported that the hands-on training during the simulation was more helpful than the departmental PAPR demonstration, reinforcing the effectiveness of hands-on training in minimizing errors during the donning and doffing process.

Residents showed the highest proficiency with interpersonal communication and with application of lung protective ventilation strategies (Table 1). Interpersonal communication skills were evaluated through interactions with the ICU attending, the ICU nurse, the patient, and the patient's family member. Although most residents were comfortable speaking with the anxious patient, some were unfamiliar with rapidly evolving COVID-19 hospital-wide policies. Participants without knowledge of system-wide policies were unable to reassure the family member, leaving the family member with unanswered questions. During the pandemic, telephone and virtual communication was integral in connecting family members and patients, who were often intubated and sedated. Interpersonal communication was explored in depth during the debrief sessions.

The integration of the ICU ventilator was essential because of the possibility of UCLA anesthesiologists providing ICU coverage during the pandemic. The ASL 5000 breathing simulator created high airway pressures commonly seen with ARDS, allowing participants to be hands-on with a ventilator commonly seen in the ICU. However, as COVID-19 cases stabilized in California, most new graduates (71%) did not find it necessary to utilize ARDSnet 3 months later in independent practice.

Confidence assessment scores expectedly improved immediately after the simulation. Knowledge assessment scores showed an average 38% improvement on the postsimulation questionnaire, with only a 6% improvement 3-months postsimulation. The knowledge assessment focused on concepts that may not be necessary competencies in the clinical care of actual COVID-19 patients. For example, it is unlikely that a provider who participates in the care of a COVID-19 patient will need to recall the filtration efficiency of N95 respirators or ARDSnet from memory. So, even though 48% of participants had taken care of a COVID-19 patient at the 3-months follow-up, their scores were no better when compared to those who had not taken care of COVID-19 patients. In fact, participants who had not taken care of COVID-19 patients actually scored higher in the 3-months postsimulation questionnaire (13% vs. −1%). Additionally, community cases of COVID-19 decreased between the time of intervention and the 3-months follow-up (September 2020), so participants may have had less opportunity to apply knowledge concepts. If they practiced more frequently and closer to the time of intervention, there may have been more significant 3-months follow-up data. Overall, knowledge scores could be improved if educational initiatives were developed to reinforce these concepts, but this likely would not have been impactful on the primary value of this simulation, which was to provide hands-on experiential training.

Participants’ perceptions of stress were lower during the simulation when compared to clinical practice. Low stress during the simulation was likely a result of simulation artifacts, as residents likely felt comfortable in a safe learning environment. Conversely, higher stress reported with actual patients is aligned with the higher mental health burden reported by residents during the early pandemic.^23^ Simulation provided the opportunity to train multiple residents simultaneously in a safe and less stressful environment, enabling experiential learning while minimizing cognitive load.

It was difficult to determine if the simulation led to a change in clinical practice due to concerns of self-reporting bias on the 3-months postsimulation questionnaire. Furthermore, many participants were working in new institutions and had variable new workplace experiences. However, of the 10 participants reporting COVID-19 patient experience, all agreed that their current management of COVID-19 patients was directly informed by the simulation training. Overall, participants reported an improvement in confidence levels and attributed changes in clinical practice 3 months after participating in the simulation, suggesting that knowledge from the simulation may have effectively translated to the clinical setting.

One strength of the study design is the use of a 3-months postsimulation questionnaire. This questionnaire allowed us to explore potential changes in clinical practice attributable to the simulation. Another strength is the reinforcement of evidence-based protocols during an active pandemic. Simulation trainees gained confidence in newly acquired skills that required psychomotor practice, such as donning and doffing with PPE. These skills would have been difficult to practice remotely, highlighting the importance of hands-on simulation training with just-in-time learning. The simulation also allowed us to improve our safety protocols to enable simulation training during a pandemic.

One limitation of the study is that this is a single-site simulation without a control group, driven by the goal to train as many PGY 4 anesthesiology residents as possible. Furthermore, due to the urgency to expose residents to PPE training, participants worked in groups of two. This may have been a limitation, but it also provided training in teamwork while wearing full PPE. Lastly, use of both N95 respirators and PAPRs throughout the simulation was due to inventory constraints.

In conclusion, we developed a high-fidelity simulation that provided the opportunity for anesthesiology residents to practice and prepare for COVID-19 patient care. While many training programs resorted to remote learning, we conducted this live simulation training safely during an ongoing pandemic in order to fill knowledge gaps that became apparent when COVID-19 intubations became common practice. We plan on integrating this simulation as part of our regular residency training program and hope to collaborate with other institutions to study the effects of repeated and longitudinal training on clinical practice. As the COVID-19 pandemic continues to evolve or as new emerging infectious diseases arise, using simulation to practice evidence-based protocols can have a positive impact on both the safety of HCPs and the quality of patient care.

## Appendices


Simulation Case Template.docxDonning and Doffing Recommendations.docxQuestionnaires and Knowledge Checks.docx
*All appendices are peer reviewed as integral parts of the Original Publication.*
