# Supplementary material for: Implementing COVID-19 Simulation Training for Anesthesiology Residents
Source: MedEdPORTAL. 2022 Jan 31;18:11215. doi: 10.15766/mep_2374-8265.11215 (PMC8801548; doi:10.15766/mep_2374-8265.11215)
Supplement: Supplementary file 1 — Simulation Case Template.docxDonning and Doffing Recommendations.docxQuestionnaires and Knowledge Checks.docx [file mep_2374-8265.11215-s001.zip › C. Questionnaires and Knowledge Checks.docx]

Appendix C: Pre-Simulation, Post-Simulation, and Three-Months Post-Simulation Questionnaires and Knowledge Checks

Pre-Simulation Questionnaire

1. How confident are you with donning and doffing with level 2 PPE?

- Not at all (1)
- Slightly (2)
- Somewhat (3)
- Very (4)
- Extremely (5)

1. How confident are you with the ventilatory management of patients with acute respiratory distress syndrome (ARDS)?

- Not at all (1)
- Slightly (2)
- Somewhat (3)
- Very (4)
- Extremely (5)

1. How confident are you with the airway management of patients with an emerging infectious disease, such as SARS-CoV-19?

- Not at all (1)
- Slightly (2)
- Somewhat (3)
- Very (4)
- Extremely (5)

1. To the best of your recollection, how many patients have you taken care of with an emerging infectious disease, such as SARS-CoV-19?

- 0
- 1-3
- 4-6
- 7-9
- 10+

Post-Simulation Questionnaire

1. After completing this simulation, how confident are you with donning and doffing with level 2 PPE?

- Not at all (1)
- Slightly (2)
- Somewhat (3)
- Very (4)
- Extremely (5)

1. After completing this simulation, how confident are you with the ventilatory management of patients with acute respiratory distress syndrome (ARDS)?

- Not at all (1)
- Slightly (2)
- Somewhat (3)
- Very (4)
- Extremely (5)

1. After completing this simulation, how confident are you with the airway management of patients with emerging infectious disease, such as SARS-CoV-19?

- Not at all (1)
- Slightly (2)
- Somewhat (3)
- Very (4)
- Extremely (5)

1. The simulation was a valuable learning experience.

- Strongly disagree (1)
- Disagree (2)
- Neither agree nor disagree (3)
- Agree (4)
- Strongly agree (5)

1. The simulation was difficult and/or stressful.

- Strongly disagree (1)
- Disagree (2)
- Neither agree nor disagree (3)
- Agree (4)
- Strongly agree (5)

1. Do you feel that you will change your future clinical practice based on this simulation? If so, what will you change? ________________
2. Please provide additional comments and feedback to improve this simulation experience. ________________

Three-month Post-Simulation Questionnaire

Dear recent graduates,

You participated in a COVID-19 airway simulation session 3 months ago. Your involvement allowed our team to evaluate the utility of simulation-based education for hands-on PPE training, identify workflow issues with COVID-19 airway/ventilator management, and identify stressors during the simulation which may affect patient care.

Please fill out the following 5-10 minute questionnaire. Whether you are working in private practice, academics, or are currently in fellowship, your response is invaluable and will help us to refine our simulation session for future residency classes. As always, we appreciate your hard work. Your responses to this survey are strictly confidential. Thank you!

1. Where do you currently work?

- Private practice
- Academic center
- Fellowship (please specify which type): ________________

1. Have you been involved in the care of a COVID-19 positive or person under investigation (PUI) since the completion of residency?

- Yes
- No

If yes, answer the following questions:

How many COVID-19 positive or PUI patients have you taken care of since completion of residency?

- 0
- 1-3
- 4-6
- 7-9
- 10+

Please indicate your agreement with the following three statements:

|  | Strongly disagree (1) | Disagree (2) | Neither agree nor disagree (3) | Agree (4) | Strongly agree (5) |
| --- | --- | --- | --- | --- | --- |
| My current management of COVID+ or PUI patients was directly informed by what I learned from the simulation session. |  |  |  |  |  |
| The principles of PPE taught during the UCLA simulation session are similar to my institution's current practices. |  |  |  |  |  |
| I find the management of COVID-19 or PUI patients to be stressful. |  |  |  |  |  |

1. Have you had additional training on managing COVID patients since the simulation training?

- Yes
- No

If yes, please indicate the type of training you had: ________________

1. Have you had to apply principles of ARDSnet ventilator management in your new institution?

- Yes
- No

If yes, please indicate your agreement with the following three statements:

|  | Strongly disagree (1) | Disagree (2) | Neither agree nor disagree (3) | Agree (4) | Strongly agree (5) |
| --- | --- | --- | --- | --- | --- |
| My current practice of ARDSnet ventilator management was directly informed by what I learned from the UCLA simulation session. |  |  |  |  |  |
| The principles of ARDSnet taught during the UCLA simulation session were similar to my institutions current practices. |  |  |  |  |  |
| I find ARDSnet ventilator management to be stressful. |  |  |  |  |  |

1. Please identify the respiratory equipment that your institution uses in the care of COVID-19+ or PUI patients (Select all that apply).

- N-95 masks
- Elastomeric half- or full facepiece air purifying respirators
- Powered Air Purifying Respirators (PAPRs)

1. Please indicate your agreement with the following statements, if applicable:

|  | N/A | Strongly disagree (1) | Disagree (2) | Neither agree nor disagree (3) | Agree (4) | Strongly agree (5) |
| --- | --- | --- | --- | --- | --- | --- |
| The UCLA simulation center PAPR hands-on session was more helpful and applicable than the departmental PAPR demonstration. |  |  |  |  |  |  |
| I have been able to teach the knowledge and skills from the simulation session to my peers and/or trainees. |  |  |  |  |  |  |

Knowledge-Based Multiple-Choice Questions and Answers (Given pre-simulation, post-simulation, and three-months post-simulation)

1. According to the NIH NHLBI ARDSnet protocol, what should your initial tidal volume be set to?
2. 4 ml/kg IBW
3. 5 ml/kg IBW
4. 6 ml/kg IBW
5. 7 ml/kg IBW
6. 8 ml/kg IBW
7. According to the NIH NHLBI ARDSnet protocol, what should your target goals be for PaO2 and SpO2:
8. PaO2 50-75mmHg, SpO2 88-95%
9. PaO2 55-80mmHg, SpO2 88-95%
10. PaO2 60-85mmHg, SpO2 88-95%
11. PaO2 65-90mmHg, SpO2 88-95%
12. PaO2 70-95mmHg, SpO2 88-95%
13. According to the NIH NHLBI ARDSnet protocol, what should your target goals be for plateau pressures:
14. 20-25cm H2O
15. 25-30cm H2O
16. 30-35cm H2O
17. 35-40cm H2O
18. 40-45cm H2O
19. Which of the following is the correct order for donning the minimum level 2 PPE with PAPR:
20. Bouffant, PAPR, gloves, gown, gloves
21. Bouffant, N95, PAPR, gloves, gown, gloves
22. Bouffant, N95, PAPR, gloves, gown, gloves, shoe covers
23. Tyvek hood, N95, PAPR, gloves, gown, gloves
24. Tyvek hood, N95, PAPR, gloves, gown, gloves, shoe covers
25. Which of the following is the correct order for doffing the minimum level 2 PPE with PAPR (hand sanitizer between each step):
26. Doff gloves, doff gown, leave room, doff and clean PAPR with face shield, unplug and clean PAPR battery and cord, doff bouffant, doff gloves
27. Doff gloves, doff gown, leave room, unplug and clean PAPR battery and cord, doff and clean PAPR with face shield, doff N95, doff bouffant, doff gloves
28. Doff gloves, doff gown, leave room, doff PAPR face shield, doff gloves, unplug and clean PAPR battery and cord, doff and clean PAPR, doff N95, doff bouffant
29. Doff gloves, doff gown, leave room, doff PAPR face shield, doff gloves, don new gloves, unplug and clean PAPR battery and cord, doff and clean PAPR, doff N95, doff bouffant, doff gloves
30. Doff gloves, doff gown, leave room, doff PAPR face shield, doff gloves, don new gloves, unplug and clean PAPR battery and cord, doff and clean PAPR, doff bouffant, doff gloves
31. What is the **minimum** recommended PPE for performing an aerosolizing procedure for a patient with **droplet** precautions?
32. Gown, gloves, face mask
33. Gown, gloves, N95, PAPR
34. Gown, gloves, N95, eye protection
35. Gown, gloves, PAPR, tyvek hood
36. Gown, gloves, N95, tyvek hood
37. What is the **minimum** recommended PPE for performing an aerosolizing procedure for a patient with **airborne** precautions?
38. Gown, gloves, face mask
39. Gown, gloves, N95, PAPR
40. Gown, gloves, N95, eye protection
41. Gown, gloves, PAPR, tyvek hood
42. Gown, gloves, N95, tyvek hood
43. How efficient are N95 masks in filtering out particles?
44. Filter at least 95% of particles <0.3 microns
45. Filter at least 95% of particles <0.5 microns
46. Filter at least 99.97% of particles <0.3 microns
47. Filter at least 99.97% of particles <0.5 microns
48. Filter at least 99.99% of particles <0.5 microns
49. How efficient are PAPRs with HEPA filters in filtering out particles?
50. Filter at least 95% of particles <0.3 microns
51. Filter at least 95% of particles <0.5 microns
52. Filter at least 99.97% of particles <0.3 microns
53. Filter at least 99.97% of particles <0.5 microns
54. Filter at least 99.99% of particles <0.5 microns
55. Awake proning is recognized as an early measure to improve oxygenation in COVID + patients because:
56. There is an increase in heterogeneity in alveolar distribution
57. Studies with awake proning show lower FiO2 has been necessary to treat hypoxemia
58. Proning helps with the increased airway resistance associated with COVID + patients
59. Closing capacity decreases with proning
60. There is a decrease in VQ mismatch

**Answers:**

1. E
2. B
3. B
4. A
5. E
6. C
7. C
8. A
9. C
10. E
