# Supplementary material for: Implementing COVID-19 Simulation Training for Anesthesiology Residents
Source: MedEdPORTAL. 2022 Jan 31;18:11215. doi: 10.15766/mep_2374-8265.11215 (PMC8801548; doi:10.15766/mep_2374-8265.11215)
Supplement: Supplementary file 1 — Simulation Case Template.docxDonning and Doffing Recommendations.docxQuestionnaires and Knowledge Checks.docx [file mep_2374-8265.11215-s001.zip › A. Simulation Case Template.docx]

| **Appendix A: Simulation Case Template**  **SIMULATION CASE TITLE:** Emerging Infectious Disease Airway Simulation  **AUTHORS:** Bryant Hong, MD, Christine Myo-Bui, MD, Victor Duval, MD, Yue Ming Huang, EdD, MHS  **LEARNER AUDIENCE:** Anesthesiology residents (all levels) | |
| --- | --- |
| **PATIENT NAME:** John Bruin  **PATIENT AGE:** 45-year-old  **CHIEF COMPLAINT:** Shortness of breath  **PHYSICAL SETTING:** Intensive Care Unit | |
|  | |
| **Brief Narrative Description of Case** | Two learners assume the role of anesthesiology attendings on the overnight call team. They are responsible for the evaluation of patients with respiratory distress throughout the hospital. At 0200, they are called to evaluate 45-year-old Mr. John Bruin, who was admitted yesterday for shortness of breath and coughing. The patient is COVID-19 positive by nasopharyngeal swab PCR. Since admission, the patient developed worsening shortness of breath concerning for respiratory failure and was recently transferred to the MICU. Attempts at awake prone positioning have not been effective. The learners must now work together to interview the patient, delegate tasks amongst themselves, don and doff with personal protective equipment (PPE), intubate the COVID-19 patient, manage post-induction hemodynamic shifts, and manage the ICU ventilator. They must practice interpersonal communication skills amongst themselves, the ICU nurse, the ICU attending, the patient, and the patient’s family member over the telephone. |
| **Primary Learning Objectives** | 1. Formulate and implement a plan for treating acute respiratory distress in a COVID-19 patient within a non-operative setting. 2. Manage a COVID-19 patient with acute respiratory distress syndrome. 3. Demonstrate teamwork and interpersonal communication skills with team members, the patient, and the patient’s family member. |
| **Critical Actions** | Preparation and donning in ante-room:   1. Communication of assessment and plan: Learner should review patient history and be able to recognize acute hypoxemic respiratory failure. Learner should coordinate and vocalize intubation plan with the ICU attending. Roles and tasks should be delegated. 2. Equipment collection: Learner should collect all recommended equipment prior to entering the room, including medication and backup airway equipment. Equipment should be checked. 3. Donning with PPE: Learner should choose appropriate PPE and demonstrate each step of the donning procedure by following posted cognitive aids. ICU attending will observe and provide real-time feedback as the safety monitor.   Induction and intubation in ICU patient room:   1. Introduction and assessment: Learner should introduce themselves and assess the patient. 2. Pre-oxygenation: Learner should switch out the patient’s simple face mask for a non-rebreather face mask to improve delivered FiO2 for preoxygenation. 3. Positioning: Learner should optimize patient positioning on the ICU gurney (such as removing the headboard, changing the gurney height, and positioning the patient in sniffing position). 4. Communication with patient: Learner should assuage patient anxiety with empathy and communicate care plan. 5. Communication with nurse and other learner: Orders should be clearly delegated. Call-outs and check-backs should be used to close the communication loop. 6. Preparation for possible difficult airway: Learner should have backup airway equipment readily available, such as a bougie or a video laryngoscope. 7. Intubation strategy: Learner should choose rapid sequence intubation, and induce with an IV induction agent (ketamine, etomidate, or propofol) and paralytic (succinylcholine or rocuronium). 8. Proper sequence of airway attachments: Learner should inflate the endotracheal tube cuff and attach the endotracheal tube to ventilator prior to applying positive pressure ventilation to prevent aerosolization of airborne pathogens. 9. Verification of endotracheal tube positioning: Learner should confirm endotracheal tube position (such as with end tidal CO2, chest rise, tube fogging, or ultrasound for lung sliding). 10. Sedation strategy: Learner should choose a continued sedation strategy (such as with dexmedetomidine or propofol). Nurse will prompt resident to vocalize plan.   Post-intubation management in ICU patient room:   1. Management of post-induction hypotension: Learner should start a vasopressor (phenylephrine, norepinephrine) for post-induction hypotension. 2. Management of ventilator: Learner should interpret and change ventilator settings for lung-protective ventilation in the setting of poor lung compliance and hypoxia (such as setting low tidal volumes and increasing PEEP). Learner should interpret and change ventilator settings with given arterial blood gas values. 3. Communication of plan with nurse: Learner should communicate with the nurse regarding goals and plan of care. 4. Doffing part 1: Learner should leave the room once the patient is stable, by doffing appropriately. Nurse will call out need to complete specific steps for doffing if learner does not do so themselves.   Doffing and debriefing in ante-room:   1. Doffing part 2: Learner continues to demonstrate appropriate doffing procedure outside of the ICU room. 2. Handoff report: Learner should provide a verbal sign-out to the ICU attending. 3. Family update: Learner should communicate with the patient’s family member over the phone, providing updates and answering questions with empathy. |
| **Learner Preparation or Prework** | Prior to the simulation, all residents should have completed mandatory N-95 respirator fitting, Powered Air Purifying Respirators (PAPRs) demonstration (observation only), and a perioperative donning and doffing training session with N-95 respirators, eye protection, head coverings, gowns, and gloves.  Learner should be provided with the patient HPI and case stem on the day of simulation. |

| Initial Presentation | | | |
| --- | --- | --- | --- |
| **Initial Vital Signs** | HR: 111 BPM  BP: 125/82 (96)  T: 38.6C/103.3F  RR: 30  SpO2: 87% on 15LPM facemask | | |
| **Overall Setting and Appearance** | 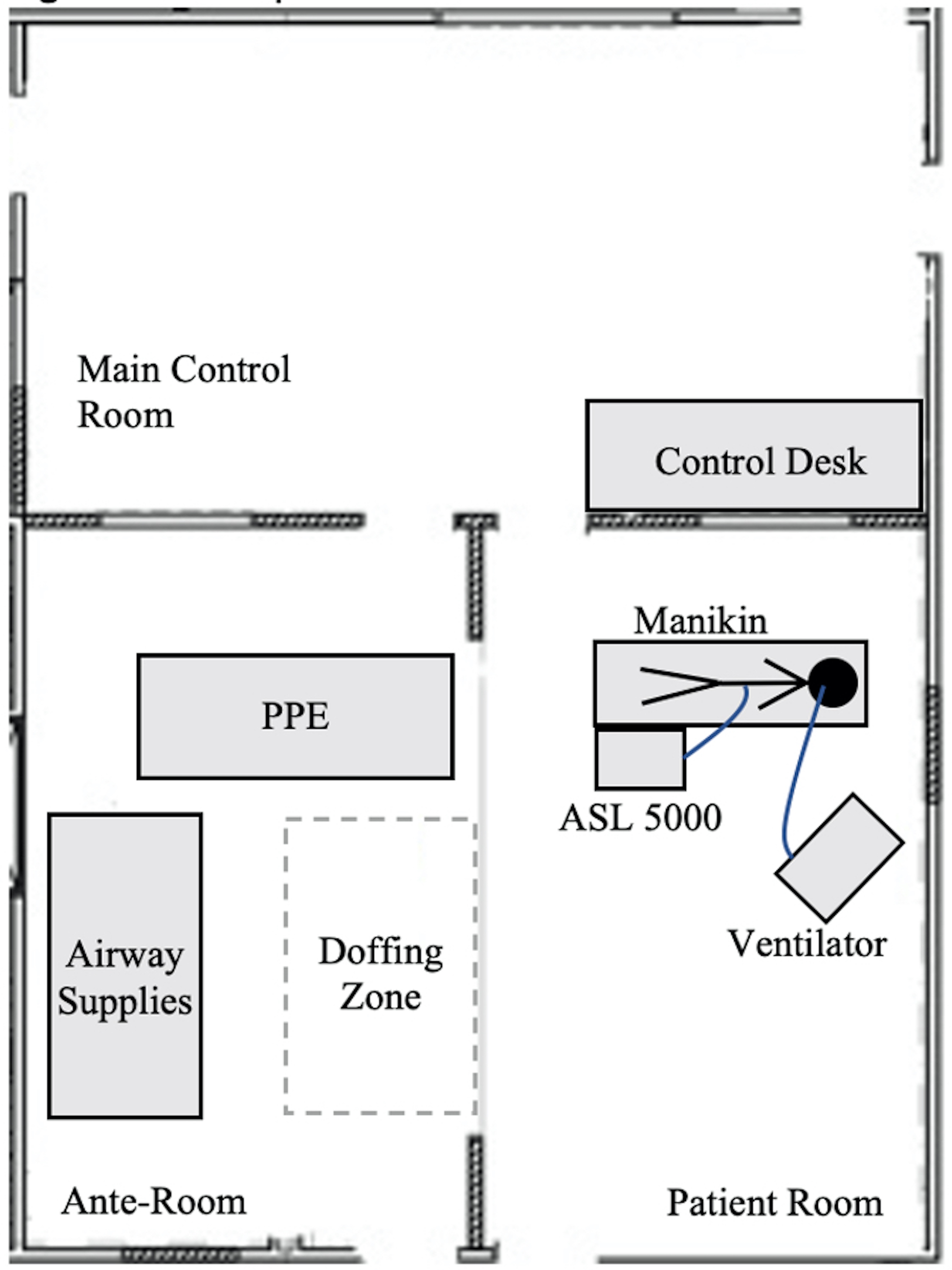This live simulation takes place at the UCLA Simulation Center, a fully staffed medical training facility. The setting is an ICU room with a conjoined ante-room.  The learners start in the ante-room, which includes PPE (gloves, gowns, eye protection, head coverings, N-95 respirators, and PAPRs), airway equipment (handles and blades for direct laryngoscopy, a video laryngoscope), and commonly used anesthetic medication. Here, they meet the ICU attending and are able to collect equipment/medication, and don/doff PPE.  In the ICU patient room, there is a computerized full-body manikin patient (Laerdal SimMan 3G, Norway), a Servo-i ventilator (Maquet, Germany), and an ASL 5000 breathing simulator (IngMar Medical, USA). The manikin is laying supine on a gurney, with a single 20 gauge peripheral IV connected to a normal saline 0.9% 1L bag, a blood pressure cuff, a 5-lead ECG, and a pulse oximeter. The manikin patient is awake, anxious, and presenting with acute hypoxemic respiratory failure while wearing a simple face mask set at 15LPM O2. The gurney is lowest to the ground with the guard rails up.  Figure: Set-up of simulation at UCLA Simulation Center. Author owned image.  From a control room, instructors observe the learners, control the computerized manikin and ASL 5000 breathing simulator, and manipulate vitals and ventilatory settings. | | |
| **Standardized Participants** | In-person actors:   - ICU attending (played by anesthesiology instructor): Role is to observe donning/doffing as the safety monitor, ask for sign-out at the end of the case, and ask the learner to talk to the patient’s family member on the phone.   - Introduction: “Thank you for coming to evaluate the patient. I am the ICU attending. SpO2 is 87% and down trending on 15LPM facemask. Our nurse is already inside and can help you from there. Here is our crash cart with airway supplies. Feel free to ask us for anything else once you’re inside the room.”   - Donning/doffing: “This is the PPE that we have available. Please don your PPE and I will observe.” Observe the donning/doffing process and point out anything the learner does incorrectly. “You put on the __ before __.”   - Sign-out: “I was busy next door with another patient. How is everything going?”   - Ask the learner to talk to family member: “I have John’s family member on the phone, can you talk to them?” - Embedded “confederate” nurse (played by simulation operator): Role is to assist the learner in the ICU room and prompt for verbalization of thoughts.   - Introduction: “Hi, I’m the nurse, __. I just started this month and this is my first COVID patient. How can I help?”   - With desaturation: Mention the SpO2 at 70% and 60%. At 70% should say, “Patient does not look responsive.”   - After intubation: “Would you like an ABG?” If yes, present the ABG as pH 7.17, PaCO2 54, HCO3 22, PaO2 65.   - If sedation was not started after intubation: “Looks like the patient is waking up. What sedation would you like to start?”   - If vasopressor was not started for hypotension: “Can the patient’s blood pressure stay this low?”   - Before leaving room: “What is the plan?” “Don’t forget to doff your ____.”   Actors over audio or video:   - Manikin Patient (played by simulation operator): Role is to be anxious and concerned prior to intubation.   - "Am I going to die?"   - "I have always been fine, but when my wife was in the hospital for 3 weeks, I was always at her bedside.... Why can't she be here? I need to see my wife and kids... I don't want to die alone."   - “Can I call my wife to let her know what’s going on?” – If the learner attempts to connect with the wife, patient will start decompensating. - Family member (played by simulation operator): The patient’s family member will call into the room by phone to ask for an update on the patient at the end of the scenario. The last time the family member saw the patient was in the emergency department last night when he was admitted. The family member was unable to come up with him due to hospital-wide policy. The family member has since stayed up all night, waiting near the hospital for an update.   - “Can you help me out? I haven’t heard from John since he was admitted last night! I want to know what is going on.”   - “I need to see John, now! What is your policy on visitors? This is completely unacceptable. I’m not sick, I’m not coughing, I just need to see him.” | | |
| **HPI** | Mr. John Bruin is a 45-year-old male with PMH of obesity (BMI 36.6) and type II diabetes who is presenting with shortness of breath and coughing. Patient initially noted fever and coughing yesterday after getting home from work. Symptoms progressively worsened and he now feels shortness of breath. Patient decided to drive to the emergency department with his family member yesterday evening. Patient denied any recent travel in past 14 days and stated he has not been in close contact with a confirmed or suspected case of COVID-19 in the past 14 days.  Review of Systems (if asked):  Constitutional symptoms — fever, lethargy  Cardiovascular — palpitations, no chest pain  Respiratory — shortness of breath, dry cough, sore throat, rhinorrhea  Gastrointestinal — No nausea, vomiting, diarrhea  Genitourinary — No dysuria  Musculoskeletal — generalized myalgia  Integumentary — No rash  CXR: peripheral basilar predominant airspace disease  Chest CT: peripheral based nodular to confluent ground glass opacities | | |
| **Past Medical/Surgical History** | **Medications** | **Allergies** | **Family History/Social** |
| PMH:  Obesity (BMI 36.6)  Type II diabetes  PSH:  None | Metformin 500mg QD | No known drug allergies | Works as engineer. Alcohol on occasion, marijuana use daily. |
| **Physical Examination** | | | |
| **General** | Appropriate for stated age, well groomed, obese. | | |
| **HEENT** | Mallampati III, thyromental distance 5cm, pupils equal, round, reactive to light and accommodation, EOMI, MMM. | | |
| **Neck** | Short neck, no LAD. | | |
| **Lungs** | Bilateral rhonchi with decreased breath sounds, no accessory muscle use. | | |
| **Cardiovascular** | Sinus tachycardia, normal S1 and S2, no M/R/G. No JVD. | | |
| **Abdomen** | Normoactive bowel sounds. Soft, flat, non-tender, and non-distended. | | |
| **Neurological** | AOx3 to self, date, location. | | |
| **Skin** | Warm, dry, well-perfused. No rashes or other lesions. | | |
| **Psychiatric** | Anxious. | | |

| Instructor Notes - Changes and CASE Branch Points | | |
| --- | --- | --- |
| **Intervention / Time point** | **Change in Case** | **Additional Information** |
| 3 minutes have passed in the ICU patient room, or if learner tries to contact/call the patient’s family member per his request, whichever occurs first. | SpO2 begins decreasing slowly until 60% over 3 minutes. Patient becomes unresponsive at 70%, eyes close. | Nurse alerts the provider of desaturation and mentions the SpO2 at 70% and 60%. At 70%, the Nurse should say, “Patient does not look responsive.” |
| Learner asks for additional equipment or staffing. |  | Nurse responds with the following:   - Non-rebreather face mask, bag-valve mask, video laryngoscope: Retrieves for learner - High flow nasal cannula, BIPAP: “We do not have any available” - Respiratory therapist: “RT is busy with another patient, you’ll have to set up the ventilator yourself” |
| Learner induces anesthesia (with any agent). | Apnea occurs 5 seconds after induction, with the subsequent development of hypoxia SpO2 50-60% within 10 seconds if intubation has not yet occurred. | Nurse administers medication if asked by learner. |
| Learner successfully intubates the patient. | SpO2 improves to 89-92% when successfully ventilating (by any means from BVM, LMA, or ETT). Blood pressure drops to 70s/30s. | Nurse asks if the learner would like an ABG. If yes, present the ABG as pH 7.17, PaCO2 54, HCO3 22, PaO2 65. |
| Learner attaches the endotracheal tube to the ventilator. | Initial hemodynamics:   - Peak pressures: 38 - Plateau pressures: 32 - SpO2 89-92% | ICU attending can prompt the learner to address high airway pressures and hypoxia. If the learner shows appropriate lung protective choices (lower TV, higher RR, high PEEP), pressures and oxygenation will improve to:   - Peak pressures: 30 - Plateau pressures: 25 - SpO2 should reach 95% |
| Nurse presents the ABG as pH 7.17, PaCO2 54, HCO3 22, PaO2 65. | Learner should increase respiratory rate and increase PEEP if possible. | ICU attending can prompt the learner to address ventilatory settings based on ABG findings. If asked, repeat ABG shows: pH 7.20, PaCO2 40, HCO3 22, PaO2 95 |
| Learner chooses continued sedation strategy. | Patient does not buck/develop high peak airway pressures after 5 minutes. | Nurse will hang the medication for sedation. |
| Learner chooses vasopressor for post-intubation hypotension. | Blood pressure improves to 100s/60s. | Nurse will hang the vasopressor of choice. If pressor not chosen, the nurse can ask if the blood pressure is appropriate. |

**Ideal Scenario Flow**

The learners enter the ante-room at the start of the simulation. They receive sign-out from the ICU attending of the COVID-19 patient in respiratory distress. They can ask follow-up questions during this time. They will then delegate roles for intubation, collect necessary intubating equipment and medication, and successfully demonstrate donning of PPE. During this time, the patient and vitals can be easily seen through the ICU room windows, and the learners can instruct the ICU nurse in the room to assist with tasks (such as preoxygenating the patient with a non-rebreather mask rather than the simple facemask, or positioning the patient for intubation). Once inside the ICU room, learners will assess and address the anxious patient’s concerns and requests. SpO2 will drop to 60% over a 3-minute period, during which time the learners will position themselves around the gurney. Learners will perform a rapid sequence intubation with the placement of the endotracheal tube and cuff inflation prior to ventilation. They will confirm endotracheal positioning by listening for bilateral breath sounds. They will then connect the endotracheal tube to the ventilator. They will need to start a vasopressor (such as phenylephrine or norepinephrine) for post-induction hypotension, and a maintenance infusion for continued sedation while mechanically ventilated (such as propofol or dexmedetomidine). The ventilator and vitals will show signs of poor airway compliance and hypoxia, concerning for ARDS, which should be treated with some changes on the ventilator, including decreasing the tidal volume, increasing the respiratory rate, and increasing the PEEP. The nurse will read out an ABG showing respiratory acidosis. The learners should increase the respiratory rate on the ventilator. Now that the patient is stabilized, they will doff part of their PPE and exit the ICU room. In the ante-room, they will finish doffing their PPE, provide a verbal sign-out to the ICU attending, and update the patient’s family member over the phone.

**Anticipated Management Mistakes**

1. Failure to prepare for a possible difficult airway: Some learners forgot to prepare or identify backup airway equipment, such as a bougie or video laryngoscope, prior to entering the ICU patient room.
2. Time-consuming or incorrect donning and doffing with PAPR: Most learners required an introduction to and significant assistance with donning and doffing with PAPR. Most learners had no experience with PAPR until the time of the simulation, sometimes leading to inappropriate handling of PAPR, inadequate equipment checks (such as a failure to interpret filter and battery light status indicators, accidental power cord disconnection), and time-consuming periods of donning and doffing.
3. Inefficient delegation of tasks: Some learners did not recognize the essential role of the ICU nurse who was in the room with the patient. During the donning and doffing process, the learners could have asked the nurse to assist with time-consuming tasks, such as placing monitors, patient positioning, and preoxygenation.
4. Failure to improve preoxygenation: Many learners did not switch out the simple facemask for a non-rebreather face mask to improve delivered FiO2 for preoxygenation.
5. Failure to verify endotracheal tube positioning: Many residents did not verify endotracheal tube positioning. This was likely multifactorial from wearing full gown PPE and preoccupation with ventilator management.
6. Failure to address post-intubation hemodynamics: Some learners were late in addressing post-intubation hypotension. A prompt from the ICU nurse was occasionally necessary to address the hypotension. Learners occasionally forgot to start an infusion for continued sedation, which we addressed by waking up the manikin.
