# Supplementary material for: Implementing COVID-19 Simulation Training for Anesthesiology Residents
Source: MedEdPORTAL. 2022 Jan 31;18:11215. doi: 10.15766/mep_2374-8265.11215 (PMC8801548; doi:10.15766/mep_2374-8265.11215)

Appendix B: University of California, Los Angeles, Department of Anesthesiology and Perioperative Medicine COVID-19 Task Force Donning and Doffing Recommendations for Powered Air Purifying Respirator and Tyvek Hood (version 4/28/20). Written by Emily Methangkool, MD, MPH, used with permission.


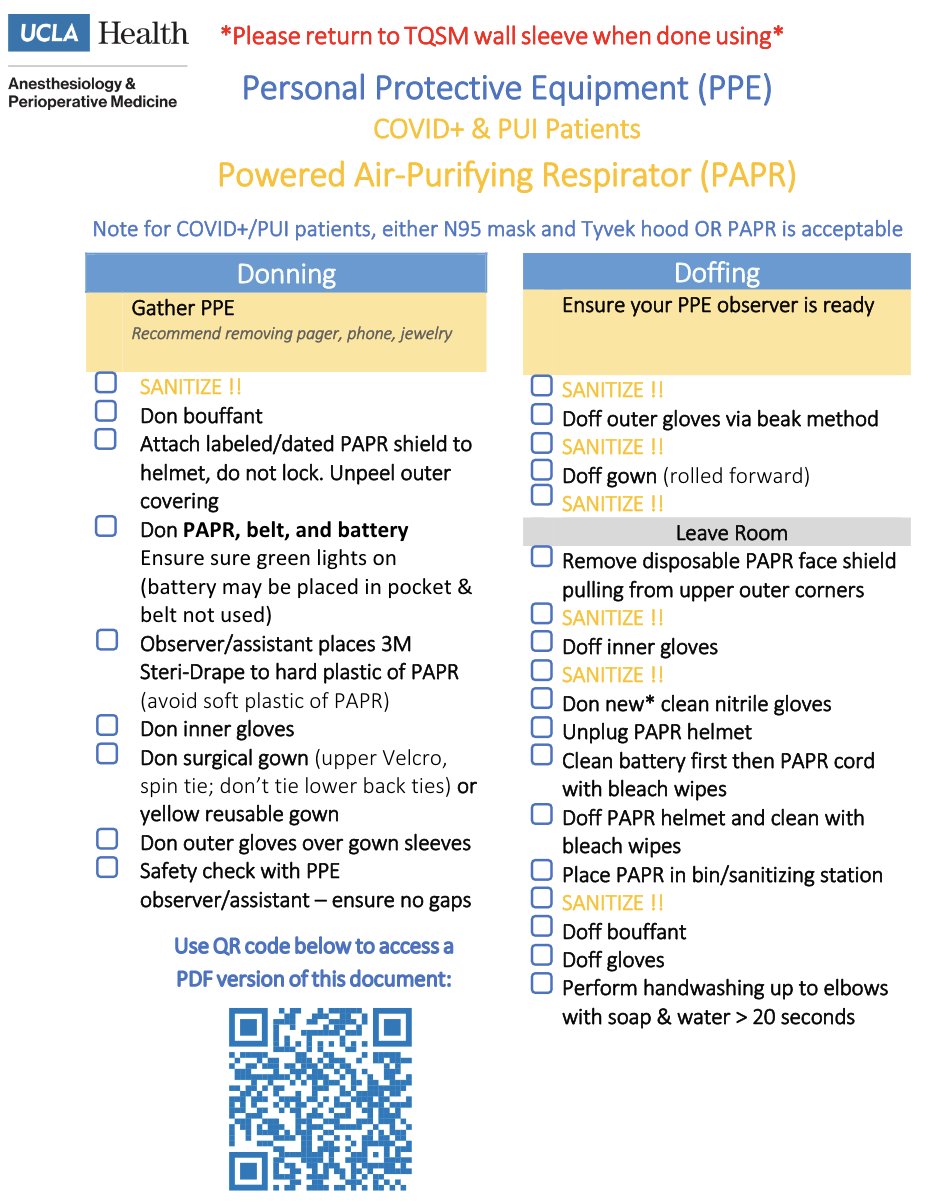


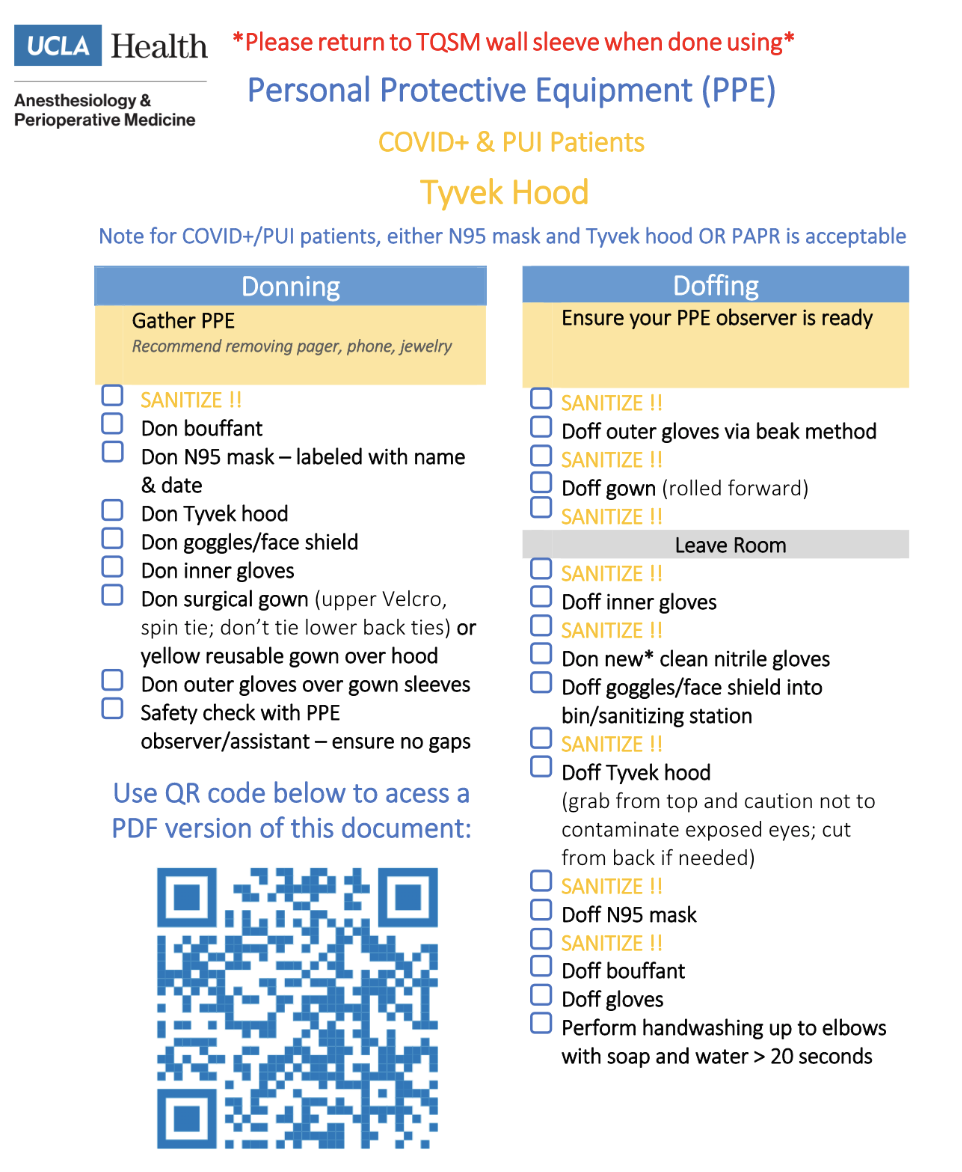

Supplement: Supplementary file 1 — Simulation Case Template.docxDonning and Doffing Recommendations.docxQuestionnaires and Knowledge Checks.docx [file mep_2374-8265.11215-s001.zip › B. Donning and Doffing Recommendations.docx]
